# Impact of changing the surgical team for wound closure on surgical site infection: A matched case-control study

**DOI:** 10.1371/journal.pone.0241712

**Published:** 2020-11-05

**Authors:** Lilian Salm, Dimitri Chapalley, Stéphanie Fabienne Perrodin, Franziska Tschan, Daniel Candinas, Guido Beldi

**Affiliations:** 1 Department of Visceral Surgery and Medicine, University Hospital Bern, Bern, Switzerland; 2 Institute of Work and Organizational Psychology, University of Neuchâtel, Neuchâtel, Switzerland; Royal Prince Alfred Hospital, AUSTRALIA

## Abstract

**Background:**

Wound closure is performed at the end of the procedure, when the attention of the surgical team may decrease due to tiredness. The aim of this study was to assess the influence of changing the surgical team for wound closure on the rate of surgical site infection (SSI).

**Methods:**

A two-armed observational monocentric matched case-control study was performed in a time series design. During the baseline period, closure of the abdominal wall was performed by the main surgical team. The intervention consisted of closure of the abdominal wall and skin by an independent surgical team. Matching was based on gender, BMI, length of surgery, type of surgery, elective versus emergency surgery and ASA score. The primary outcome was SSI rate 30 days after surgery.

**Results:**

A total of 72 patients in the intervention group were matched with 72 patients in the baseline group. The SSI rate after 30 days in the intervention group was 10% (n = 7) and in the baseline group 21% (n = 15) (p = 0.064). Redo-Surgery as result of infection (e.g. opening the wound, drainage or reoperation) was significantly higher in the baseline group (19.4% vs 2.7%; p = 0.014). Mortality, length of stay, rehospitalisation and complication rates 30 days after surgery did not differ significantly.

**Conclusion:**

Changing the surgical team for wound closure did not reduce the overall rate of SSI, but the rate of redo-surgery as a result of SSI. Despite being potentially beneficial, organizational factors are a main limiting factor of changing the surgical team for the wound closure.

**Trial registration:**

Clinicaltrial.gov NCT04503642.

## Introduction

Surgical site infections (SSI) occur in 2% to 5% patients after surgery and have a relevant impact on patient outcome and socioeconomic costs [1–5]. Risk factors for SSI can be categorized into extrinsic (procedure-related) and intrinsic (patient-related) factors [1].

Various studies have shown that the performance of the surgical team and their communication impact on SSI, in addition to the patient’s comorbidities, such as diabetes and obesity [6–8]. Surgical performance may impact on effective hemostasis, gentle handling of tissues, removal of devitalized tissue and appropriate use of drains and suture material, and thereby impact the outcome [1, 2, 9, 10].

Wound closure occurs at the end of the procedure, when the attention of the surgeons may decrease due to tiredness. We hypothesize that changing the surgical team for abdominal and wound closure improves outcome. This independent surgical team might be less tired, and would potentially interpret abdominal wall closure as their primary task. In contrast, the main team might be tired and interpret closure as a secondary task, the routine procedure at the end of a more complex and intellectually intensive operation. To address this hypothesis we changed the surgical team for wound closure, the last step of the operation, and analyzed its impact on SSI.

## Material and methods

### Patient population and data collection

This prospective matched case-control study was performed at the Department of Visceral Surgery of the University Hospital Bern, a tertiary care centre, in a time series design. During a baseline period of nine consecutive months (01.03.2016 to 30.11.2016), wound-closure was performed through the primary surgical team, as is our standard of care. Thereafter the intervention was performed during a nine consecutive months period (01.05.2017 to 31.01.2018), where abdominal wall closure was performed through a second surgical team.

Inclusion criteria were patients > 18 years of age undergoing elective or emergency laparoscopic or open abdominal surgery in our institution, performed from Monday to Friday. Patients with pre-existing SSI were excluded. The Institutional Review Board of the canton of Bern (KEK#2017–00104, KEK#161/2014) approved the study and participants were included if they gave written general consent. The data was stored in a database system (REDCap database) and checked for accuracy and completeness.

A 1:1 case-control study design was used to compare the patients in the intervention group with the patients in the baseline group. Patients were matched according to type of surgery (hernia, upper GI, hepatobiliary, colorectal, kidney transplantation and other surgeries), gender, Body Mass Index (BMI)(+/-2.5kg/m2), duration of surgery (+/-30min), elective versus emergency surgery and American Society of Anesthesiologists (ASA) physical status classification (ASA > or ≤ 2).

This study was conducted using the TREND checklist for non-randomized studies [11].

### Intervention

During the baseline period, closure of the abdominal wall was performed by the main surgical team, the same team that performed the whole surgery. The intervention consisted in the closure of the abdominal wall and skin by a second surgical team which included a board-certified surgeon and a resident. The wound closure in the baseline group was done by the primary surgical team that includes a board-certified surgeon.

Standard methods for the prevention of SSI were applied to all patients, including timing and duration of antibiotic prophylaxis [12–15]. Hairs were removed from the operating field using clippers immediately before the operation [16, 17]. Skin disinfection was applied 3 times using chlorhexidine for the abdominal wall and a povidone-iodine–based disinfectant for the inguinal and perineal region. Single-use sterile drapes were used in all patients [18]. All members of the surgical team wore surgical caps, surgical masks, two layers of sterile gloves and a one-way surgical gown [19, 20]. Laparotomy incision closure in both groups consisted of a running suture with a 2:0 PDS-loop. Fascia closure after laparoscopy consisted of interrupted Vicryl 2:0 stiches. Skin closure was left to the surgeon`s preference. The institutional standard contains a skin closure using interrupted sutures with polypropylene for incisions after laparoscopy, staples for incisions after liver transplantation and after emergency procedures and intracutaneous resorbable sutures for all other incisions. During the study period (2016–2018), the senior surgeons performing the different surgeries did not change.

### Endpoints and follow-up

The primary outcome of this study was the rate of SSI 30 days after surgery. SSI was defined and assessed according to the criteria developed by the Centers for Disease Control and Prevention [21–23]. Secondary outcomes were 30-day mortality, length of stay, day in intensive care unit, complication rates 30 days after surgery according Dindo-Clavien criteria [24], rehospitalisation and the treatment of SSI.

### Statistical analysis

Categorical variables were expressed as frequencies and percentages and continuous variables as median and standard deviations (SD) or mean and range (R), as appropriate. Mann–Whitney U-test were used for quantitative data. Chi-square test was used to compare dichotomous and qualitative data. A *p-*value below 0.05 was considered statistically significant. All statistical analyses were performed using SPSS Version 25.0 (IBM Corp. Released 2017. IBM SPSS Statistics for Windows, Version 25.0. Armonk, NY: IBM Corp.).

## Results

**Fig 1:**
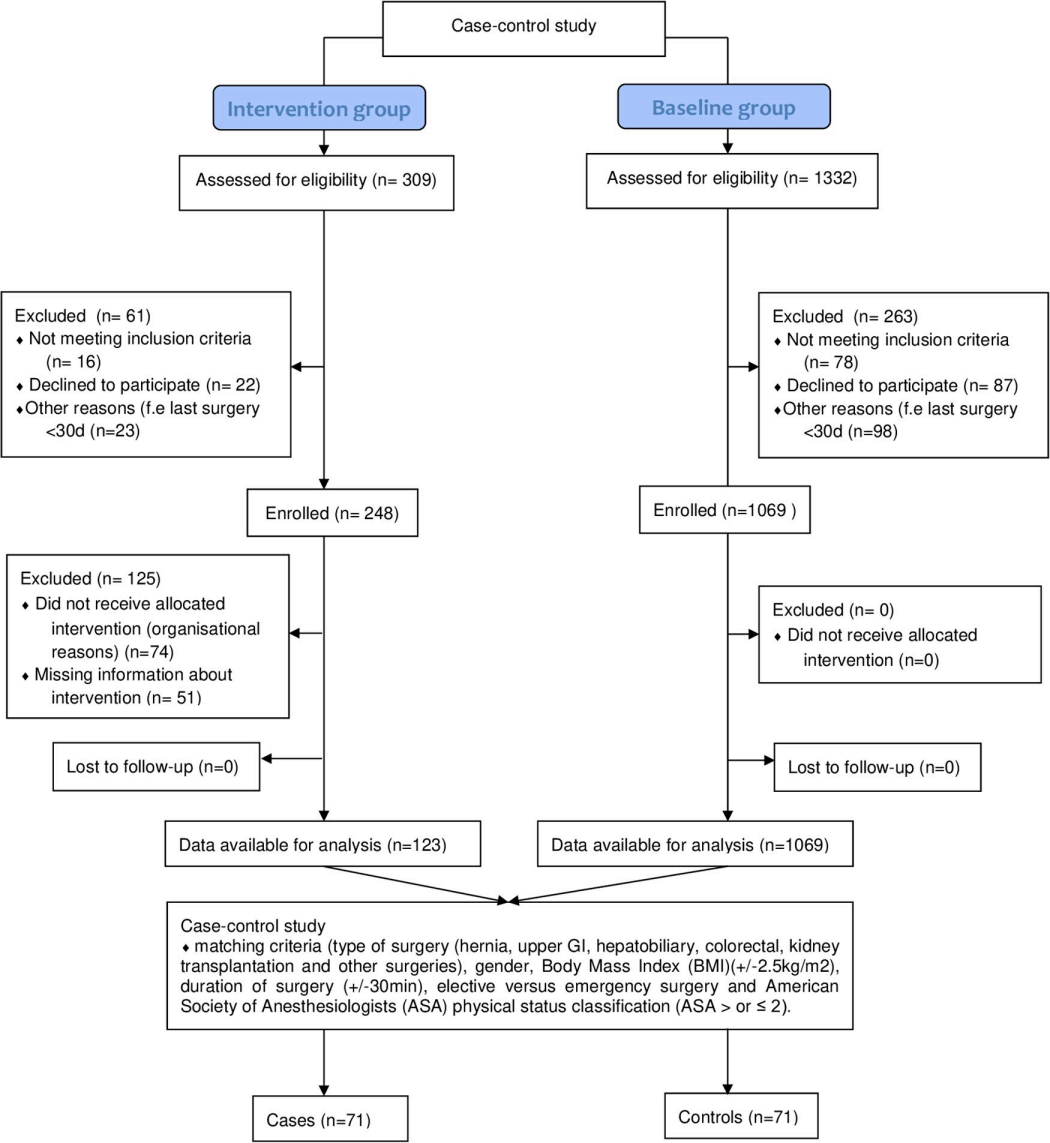
Flow diagram.

**Fig 2:**
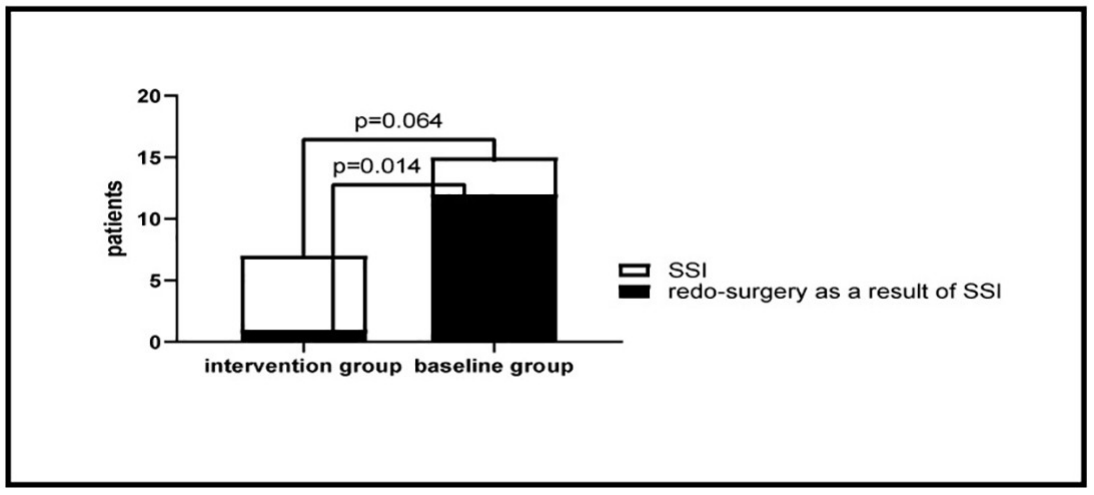
Number of SSI and redo-surgery as a result of infection in the intervention group versus the baseline group.

From March 2016 to November 2016 (9 months), 1069 patients were included in the baseline group. From May 2017 to January 2018 (9 months), 123 patients were included in the intervention group. Finally, after matching, a total of 72 pairs (144 patients) were analysed (Fig 1). Patient’s characteristics are shown in Table 1. There was no significant difference between the two groups except for previous laparotomy (61% in the baseline group versus 29% in intervention group, p<0.001). 51% of the operations were hepatobiliary, including gallbladder, liver and pancreas surgery. The SSI rate after 30 days in the intervention group was 10% (n = 7) and in the baseline group 21% (n = 15) (p = 0.064) (Table 2, Fig 2). However, there were significantly more redo-surgeries as a result of infection, such as removing suture material, drainage or reoperation, in the baseline group than in the intervention group (19.4% vs 2.7%; p = 0.014) (Table 2, Fig 2). Mortality, length of stay, rehospitalisation, day in intensive care unit and complication rates 30 days after surgery did not differ significantly between the baseline and intervention group (Table 3). The overall morbidity in the intervention group was 22.2% versus 33.3% in the baseline group (p = 0.192). There was a trend towards more severe complications (Clavien Dindo grade ≥ 3) in the baseline group (18% versus 8%, p = 0.085). We observed no fascial dehiscence in the intervention group, and one in the baseline-group. We performed a subgroup analysis including operations that lasted more than 180min. In this group, the SSI rate did not differ significantly in the intervention group (13%, n = 4) versus the baseline group (29%, n = 9) (p = 0.211) (Table 4). No adverse events occurred during the study.

**Table 1:** Patient`s characteristics.

	INTERVENTION GROUP	BASELINE GROUP	*P-*VALUE
	n = 72	n = 72	
Age, y, median (range)	64 (35-86y)	64 (26-89y)	*0*,*669*
Female sex, n (%)	28 (39%)	28 (39%)	*1*
Comorbidities			
Diabetes, n (%)	9 (13%)	15 (21%)	*0*,*180*
Chemotherapy, n (%)	8 (11%)	4 (6%)	*0*,*228*
Malnutrition, n (%)	6 (8%)	12 (17%)	*0*,*131*
Immunosuppression, n (%)	3 (4%)	3 (4%)	*1*
BMI kg/m2, median (range)	24,5 (19.2–37.9)	24,1 (18.3–38.2)	*0*,*737*
Cardiovascular disease, n (%)	11 (15%)	18 (25%)	*0*,*146*
Renal insufficiency, n (%)	10 (14%)	14 (22%)	*0*,*194*
Liver cirrhosis, n (%)	3 (4%)	4 (6%)	*0*,*698*
Previous laparotomy, n (%)	21 (29%)	44 (61%)	<*0*.*001*
ASA classification >2, n (%)	49 (68%)	49 (68%)	*1*
Type of surgery			
Elective surgery, n (%)	71 (99%)	71 (99%)	*1*
Laparoscopy n (%)	27 (38%)	27 (38%)	*1*
Mesh implantation	4 (6%)	6 (8%)	*0*,*512*
Duration of surgery in minutes, median (range)	150 (45–508)	158 (47–506)	*0*,*989*
Type of operation			
hernia, open or laparoscopic, n (%)	4 (5%)	4 (5%)	*1*
upper gastrointestinal surgery, n (%)	9 (10%)	9 (10%))	*1*
hepatobiliary surgery (incl. pancreas), n (%)	44 (51%)	44 (51%))	*1*
colorectal surgery, n (%)	6 (7%)	6 (7%)	*1*
transplantation, n (%)	2 (2%)	2 (2%)	*1*
other (Appendicitis included), n (%)	7 (%)	7 (8%)	*1*
Wound contamination level			
1 = Clean, n (%)	5 (7%)	3 (4%)	*0*,*467*
2 = Clean-contaminated, n (%)	56 (78%)	52 (72%)	*0*,*441*
3 = Contaminated, n (%)	6 (8%)	13 (18%)	*0*,*085*
4 = Infected, % (n)	5 (7%)	4 (6%)	*0*,*731*

COPD: chronic obstructive pulmonary disease, BMI: body mass index y: years, ASA: American Society of Anaesthesiology.

**Table 2:** Surgical site infection.

	INTERVENTION GROUP	BASELINE GROUP	*P-VALUE*
	n = 72	n = 72	
Overall SSI, n (%)	7 (10%)	15 (21%)	*0*,*064*
Superficial incisional SSI, n (%)	2 (3%)	1 (1%)	*0*,*102*
Deep incisional SSI, n (%)	1 (1%)	5 (7%)	*0*,*560*
Organ/Space SSI, n (%)	4 (6%)	9 (13%)	*0*,*146*
Redo-surgery as result of infection			
Intervention, n (%)	1 (3%)	12 (17%)	*0*,*001*
Removing suture material, n (%)	0 (0%)	4 (6%)	*0*,*043*
Percutaneous Drainage, n (%)	1 (3%)	5 (7%)	*0*,*095*
Reoperation, n (%)	0 (0%)	3 (4%)	*0*,*080*

**Table 3:** Outcome.

	INTERVENTION GROUP	BASELINE GROUP	*P-*VALUE
	n = 72	n = 72	
Length of hospital stay in days, median (range)	4 (1–39)	5.5 (1–29)	*0*,*317*
Intensive care unit, day, mean	1.1 (0–6)	1 (0–5)	*0*,*966*
Overall complications, n (%)	16 (22%)	24 (33%)	*0*,*192*
Clavien Dindo grade ≥ III	6 (8%)	13 (18%)	*0*,*085*
Fascia dehiscence	0 (0%)	1 (1%)	*0*,*316*
Rehospitalisation, n (%)	3 (4%)	8 (11%)	*0*,*111*
Mortality	0 (0%)	1 (1%)	*0*,*316*

**Table 4:** Subgroup analysis: Duration of operation and SSI.

	INTERVENTION GROUP		BASELINE GROUP		*P-*VALUE
	n = 72		n = 72		
Overall SSI, n (%)	7 (10%)	72	15 (21%)	72	*0*,*064*
<180 min, n (%)	3 (7%)	41	6 (15%)	41	*0*,*102*
>180 min, n (%)	4 (13%)	31	9 (29%)	31	*0*,*211*

## Discussion

To our knowledge, this is the first study that assesses the impact of changing teams during wound closure on surgical site infections. We found no difference in the primary outcome between the baseline and intervention group. There were fewer redo-surgery as a result of SSI in the intervention group, which may suggest that the SSIs in the intervention group were less severe. To what extent a causal relationship with the intervention exists remains unclear.

Wound closure occurs at the end of the procedure, when the attention of the team and especially the surgeons may decrease due to tiredness. In previous studies we have found that attention is vulnerable especially during this period [7]. Therefore, interventions aiming to decrease wound-associated complications are likely to be most efficient if performed at this time point. Team training interventions in other surgical fields have often shown limited effects [25], whereas specific interventions are more promising [26, 27]. Changing the surgical team is an intervention that seems easily applicable in everyday practice, and might reduce the rate of SSI. However, organizational aspects impairing availability and prompt recruitment of the closure team were found to be limiting factors for the implementation of the intervention. For organizational reasons, the closure team had to be composed partly of surgeons from other operations. Late arrival or unavailability of the closure team for wound closure was then the main reasons for low compliance to protocol, resulting in the small number of patients in which the intervention was actually performed.

To address this aspect of increasing surgeon fatigue towards the end of the operation, we have performed a subgroup analysis for operations that lasted longer than 180 minutes that did not reveal a significant difference. Potentially, future and larger studies may identify if this aspect as being of clinical relevance.

This analysis is subject to several limitations. Firstly, the small number of study participants. We could reach two homogenous groups solely by matching on a high number of known risk factors of postoperative wound infection, leading to the loss of 51 of the patients in the intervention group. Secondly, in our study no systematic change of gloves and gowns war performed in the baseline group and this may be a confounding factor of our study. However, the impact of changing gloves and gowns on SSI remains conflicting [6, 28–30]. Thirdly, the skin closure was left to the surgeon`s preference and consisted of staples, interrupted polypropylene sutures or subcutaneous resorbable sutures. However, because of standards on the application of these closure techniques in the institution it is likely that the different techniques are distributed not significantly different in the two groups.

In conclusion, standardized changing of the surgical team for wound closure did not reduce the overall rate of SSI, but the rate of redo-surgery as a result of SSI. Despite being potentially beneficial, organizational factors are a main limiting factor of an exchange of the surgical team for wound closure.

## Supporting information

S1 File Clinical study protocol. (DOCX)

S1 Checklist TREND statement checklist. (PDF)
