# Supplementary material for: Impact of changing the surgical team for wound closure on surgical site infection: A matched case-control study
Source: PLoS One. 2020 Nov 5;15(11):e0241712. doi: 10.1371/journal.pone.0241712 (PMC7643954; doi:10.1371/journal.pone.0241712)
Supplement: S1 File — (DOCX) [file pone.0241712.s001.docx]

Study Protocol

Impact of a specialised wound-closing

team on surgical site infections: A

prospective observational clinical trial

Short title: The CLO-T (CLOsure Team) trial

| Study Type: | Prospective controlled, two armed, observational, monocentric study |
| --- | --- |
| Study Categorisation: | A |
| Study Registration: | ClinicalTrials.gov |
| Principal Investigator: | Prof. Dr. med. Guido Beldi, Department of Visceral Surgery and Medicine, University hospital Bern (Inselspital), 3010 Bern |
|  |  |
| Protocol Version and Date: | Version 1: 18.1.2017 |

CONFIDENTIAL

The information contained in this document is confidential and the property of the principal investigator. The information may not - in full or in part - be transmitted, reproduced, published, or disclosed to others than the applicable Competent Ethics Committee(s) and Regulatory Authority(ies) without prior written authorisation from the sponsor except to the extent necessary to obtain informed consent from those who will participate in the study.

Signature Page(s)

| Study number | Study registry and registration number (Study will be registered once ethical approval is obtained) |
| --- | --- |
| Study Title | Impact of a specialised wound-closing team on surgical site infections: A prospective observational clinical trial |

The Investigator has approved the protocol version [V1.0, 18.1.2017], and confirms hereby to conduct the study according to the protocol, current version of the World Medical Association Declaration of Helsinki, ICH-GCP guidelines or ISO 14155 norm if applicable and the local legally applicable requirements.

Principle Investigator:

Prof. Dr. med. Guido Beldi, Department of Visceral Surgery and Medicine, University hospital Bern (Inselspital), 3010 Bern

| Place/Date |  | Signature |
| --- | --- | --- |

Table of Contents

[Study synopsis 5](#_Toc461176044)

[Abbreviations 9](#_Toc461176045)

[1. STUDY ADMINISTRATIVE STRUCTURE 10](#_Toc461176046)

[1.1 Sponsor, Sponsor-Investigator 10](#_Toc461176047)

[1.2 Principal Investigator 10](#_Toc461176048)

[1.3 Statistician ("Biostatistician") 10](#_Toc461176049)

[1.4 Monitoring institution 10](#_Toc461176051)

[1.5 Data Safety Monitoring Committee 10](#_Toc461176052)

[1.6 Any other relevant Committee, Person, Organisation, Institution 10](#_Toc461176053)

[2. ETHICAL AND REGULATORY ASPECTS 11](#_Toc461176054)

[2.1 Study registration 11](#_Toc461176055)

[2.2 Categorisation of study 11](#_Toc461176056)

[2.3 Competent Ethics Committee (CEC) 11](#_Toc461176057)

[2.4 Ethical Conduct of the Study 11](#_Toc461176059)

[2.5 Declaration of interest 11](#_Toc461176060)

[2.6 Patient Information 11](#_Toc461176061)

[2.7 Participant privacy and confidentiality 12](#_Toc461176062)

[2.8 Early termination of the study 12](#_Toc461176063)

[2.9 Protocol amendments 12](#_Toc461176064)

[3. Background and Rationale 13](#_Toc461176065)

[3.1 Background and Rationale 13](#_Toc461176066)

[3.2 Clinical Evidence to Date 13](#_Toc461176069)

[3.3 Explanation for choice of comparator (or placebo) 13](#_Toc461176071)

[3.4 Risks / Benefits 13](#_Toc461176072)

[3.5 Justification of choice of study population 13](#_Toc461176073)

[4. STUDY OBJECTIVES 14](#_Toc461176074)

[4.1 Overall Objective 14](#_Toc461176075)

[4.2 Primary Objective 14](#_Toc461176076)

[4.3 Secondary Objectives 14](#_Toc461176077)

[5. STUDY OUTCOMES 15](#_Toc461176079)

[5.1 Primary Outcome 15](#_Toc461176080)

[5.2 Secondary Outcomes 15](#_Toc461176081)

[5.3 Other Outcomes of Interest 15](#_Toc461176082)

[6. STUDY DESIGN 16](#_Toc461176084)

[6.1 General study design and justification of design 16](#_Toc461176085)

[6.2 Methods of minimising bias 16](#_Toc461176086)

[6.2.1 Randomisation 16](#_Toc461176087)

[6.2.3 Other methods of minimising bias 16](#_Toc461176089)

[7. STUDY POPULATION 17](#_Toc461176091)

[7.1 Eligibility criteria 17](#_Toc461176092)

[7.2 Recruitment and screening 17](#_Toc461176093)

[7.3 Assignment to study groups 17](#_Toc461176094)

[7.4 Criteria for withdrawal / discontinuation of participants 17](#_Toc461176095)

[8. STUDY INTERVENTION 18](#_Toc461176096)

[8.1 Identity of Investigational Products (treatment / medical device) 18](#_Toc461176097)

[8.1.1 Experimental Intervention (treatment / medical device) 18](#_Toc461176098)

[8.1.2 Control Intervention (standard/routine/comparator treatment / medical device) 18](#_Toc461176099)

[8.2 Compliance with study intervention 18](#_Toc461176102)

[8.3 Data Collection and Follow-up for withdrawn participants 18](#_Toc461176103)

[8.4 Trial specific preventive measures 18](#_Toc461176104)

[8.5 Concomitant Interventions (treatments) 18](#_Toc461176105)

[9. STUDY ASSESSMENTS 19](#_Toc461176106)

[9.1 Assessments of outcomes 19](#_Toc461176107)

[9.1.1 Assessment of primary outcome 19](#_Toc461176108)

[9.1.2 Assessment of secondary outcomes 19](#_Toc461176109)

[9.1.3 Assessment of other outcomes of interest 19](#_Toc461176110)

[9.1.4 Assessment of safety outcomes 19](#_Toc461176111)

[10. STATISTICAL METHODS 20](#_Toc461176113)

[10.1 Hypothesis 20](#_Toc461176114)

[10.2 Determination of Sample Size 20](#_Toc461176115)

[10.3 Statistical criteria of termination of trial 20](#_Toc461176116)

[10.4 Planned Analyses 20](#_Toc461176117)

[10.4.1 Primary Analysis 20](#_Toc461176118)

[10.4.2 Secondary Analyses 20](#_Toc461176119)

[11. QUALITY ASSURANCE AND CONTROL 21](#_Toc461176121)

[11.1 Data handling and record keeping / archiving 21](#_Toc461176122)

[11.1.1 Case Report Forms 21](#_Toc461176123)

[11.1.2 Data security and back-up 21](#_Toc461176124)

[11.1.3 Analysis and archiving 21](#_Toc461176125)

[11.1.4 Record keeping 21](#_Toc461176126)

[11.1.5 Data validation 22](#_Toc461176127)

[11.2 Monitoring and supervision procedures and data quality assurance 22](#_Toc461176128)

[11.2.1 Monitoring 22](#_Toc461176129)

[11.2.2 Supervision 22](#_Toc461176130)

[11.3 Publication policy and final report 22](#_Toc461176131)

[12. INSURANCE 22](#_Toc461176132)

[13. REFERENCES 23](#_Toc461176133)

**14. APPENDIX…………………………………………………………………………………………….……24**

14.1 Swissnoso CRF…………………………………………………………………………………………...24

14.2 Patience Information (D,F,E,I)…………………………………………………………………………..27

14.3 General Consent (D,F,E,I)……………………………………………………………………………….32

Study synopsis

| Sponsor / Sponsor-Investigator | Prof.Dr. med. Guido Beldi, Department of Visceral Surgery and Medicine, Inselspital, Bern University hospital University of Bern, 3010 Bern |
| --- | --- |
| Study Title: | Impact of a specialised wound-closing team on surgical site infections: A  prospective observational clinical trial |
| Short Title/Study ID: | CLOT trial |
| Protocol Version and Date: | Version: 1 Date: 18.01.2017 |
| Trial registration: | ClinicalTrials.gov (as soon as ethical approval is obtained) |
| Study category and Rationale | Category A: Observational study without relation to the use of drugs or medical devices. The risk for the participant is minimal and the study provides no additional strain to the participant. |
| Background and Rationale: | Surgical site infection is a frequent complication after abdominal surgery. The wound closure is done at the end of the procedure when the attention of the entire team may be affected because of tiredness and reduced attention of the surgical team.  With this study we aim to test if an exchange of the surgical team by a specialised wound closure team may reduce the impact of surgical site infection. |
| Objective(s): | To assess the influence of a specialised wound closure team on surgical site infections. |
| Outcome(s): | Primary outcome: Incidence of surgical site infections 30 days postoperatively.  Secondary outcome: mortality at 30 days, fascial dehiscence at 30 days postoperatively, postoperative complications according to Clavien-Dindo. |
| Study design: | Prospective controlled, observational, interrupted time series, monocentric study (before and after study). |
| Inclusion/Exclusion criteria: | Inclusion criteria : Patients undergoing elective or emergent laparotomy  Exclusion criteria : Preexisting surgical site infection |
| Measurements and procedures: | The control arm consists of 580 patients from the previous StOP?-study.  580 patients will be recruited in the trial arm.  Every patient has a follow up of thirty days in line with the recording of Swissnoso (national surveillance for SSI). |
| Study Intervention: | The wound closure will be done by a specialised team including one trained surgeon and one student. |
| Control Intervention | The control group consists of the study population of the StOP?-study. In this population the wound closure was done by the operation team under supervision of the main surgeon. |
| Number of Participants with Rationale: | Total of participants: 1160 (580 trial group, 580 control group). |
| Study Duration: | 2x9 months |
| Study Schedule: | Month Year of First-Participant-In:1.3.2016  Month Year of Last-Participant-Out: 30.11.2017  (baseline = 2^nd^ arm from the STOP? trial 1.3.2016 – 30.11.2016;  Intervention: 1.3.2017 – 30.11.2017) |
| Investigator(s): | Prof. Dr. med. Guido Beldi, Department of Visceral Surgery and Medicine, University hospital Bern (Inselspital), 3010 Bern |
| Study Centre(s): | University hospital Bern (Inselspital) |
| Statistical Considerations: | Sample size: The incidence of SSI after abdominal surgery in the department for Visceral Surgery and Medicine is 20%. The aim is to detect a reduction of the overall incidence rate of SSI from 20% to 14% after a follow-up of 30 days. We defined the level of significance at 5% and power of 80%, assuming normal approximation to binomial distribution requires 484 surgeries in each cell, thus 968 patients overall. In the ongoing StOP?-study we have a drop out and study refusal rate of maximal 20%. Thus, a total of 1160 participants (580 per arm) need to be included.  Duration: The average number of laparotomies from 8:00 AM to 5:30 PM from Monday to Friday per year are 720. The study schedule includes first 9 months in the control (baseline: 2^nd^ arm of the STOP? Study) and then 9 months in the intervention group.  Outcome parameters will be assessed the same way as in the ongoing StOP? Study. Thus, the results of the StOP? Study will be used as the control arm and the intervention arm will start after termination of the StOP? study. |
| Risk-Benefit statement / GCP Statement: | SSI infections are associated with increased morbidity and length of hospital stay. Patients with SSI after laparotomy or laparoscopy typically need bed-side reopening of the skin or administration of antibiotic therapy. Thus, prevention of SSI is of high interest.  The primary treatment of the patient is not affected by the trial. The change of the surgical team potentially may irritate and distract other personnel that is involved in the treatment of the patient. This may be of relevance during the initial phase of the study. The operation time may rise a little. A higher risk for the patient is not to be expected as long as communication within the surgical team assures that all relevant information is passed to the closure team. Conversely, exchange of team members is frequently done in clinical routine for both surgeons and nurses.  This study will be conducted in compliance with the protocol, the current version of the Declaration of Helsinki, the ICH-GCP or ISO EN 14155 (as far as applicable) as well as all national legal and regulatory requirements. |

Studienzusammenfassung

| Sponsor / Sponsor-Investigator | Prof.Dr. med. Guido Beldi, Department of Visceral Surgery and Medicine, Inselspital, Bern University hospital University of Bern, 3010 Bern |
| --- | --- |
| Titel: | Impact of a specialised wound-closing team on surgical site infections: A  prospective observational clinical trial |
| Abkürzung: | CLOT trial |
| Protokollversion: | Version: 1 Date: 18.01.2017 |
| Kategorie | Kategory A: Beobachtungsstudie. Die Behandlung der Patienten wird nicht verändert.. |
| Rationale | Wundinfekte sind eine häufige Komplikation nach abdominalchirurgischen Eingriffen. Der Wundverschluss erfolgt zu einem Zeitpunkt an welchem das chirurgische Team unter Umständen nach einer langen Operation müde und weniger konzentriert ist. Mit dieser Studie soll geprüft werden ob durch die Einführung von einem Wundverschlussteam die Rate an Wundinfekten gesenkt werden kann. |
| Ziel: | Prüfen ob die Inzidenz von Wundinfekten durch die Einführung eines Wundverschlussteams verändert wird. |
| Endpunkte: | Primärer Endpunkt: Wundinfekte 30 Tage postoperativ.  Sekundäre Endpunkte: Mortalität, Fasziendehiszenz, Komplikationen 30 Tage postoperativ. |
| Studiendesign: | Prospektiv kontrollierte Beobachtungsstudie (vorher – nachher Studie). |
| Ein- Ausschlusskriterien: | Einschlusskriterien: Patienten welche sich einer Laparotomie unterziehen  Ausschlusskriterien: Vorbestehender Wundinfekt |
| Prozeduren | Wundinfektsurveillance gemäss Swissnoso 30 Tage postoperativ. |
| Intervention: | Der Wundverschluss wird durch ein spezialisiertes Wundverschlussteam (1 Chirurg und 1 Student/Assistenzarzt) durchgeführt. |
| Kontrollgruppe | Studienpopulation des zweiten Armes der StOP?-Studie. In dieser Population wurde der Wundverschluss durch das primäre Operationsteam durchgeführt. |
| Anzahl Teilnehmer: | Teilnehmer 1160 (580 Intervention, 580 Kontrolle). |
| Studiendauer: | 2x9 Monate |
| Zeitplan: | Kontrollgruppe 1.3.2016 – 30.11.2016 (=2. Arm der STOP? Studie)  Intervention: 1.3.2017 – 30.11.2017 |
| Studienleiter | Prof. Dr. med. Guido Beldi, Department of Visceral Surgery and Medicine, University hospital Bern (Inselspital), 3010 Bern |
| Studienzentrum | Department für Viszerale Chirurgie und Medizin, Inselspital, Bern |
| Statistische Überlegungen: | Studienpopulation: Die Inzidenz von Wundinfekten nach abdomineller Chirurgie in der Klinik für Viszerale Chirurgie und Medizin, Inselspital Bern ist 20%. Das Ziel der Vorliegenden Studie ist zu prüfen ob durch die Intervention die Inzidenz von Wundinfekten auf 14% reduziert werden kann. Bei einer Signifikanz von 5% und einer Power von 80% ergibt sich eine Population von 484 Patienten pro Arm. Bei einer erwarteten Dropout Rate von 20% müssen 580 Patienten pro Arm eingeschlossen werden. Somit müssen gesamthaft 1160 in die Studie eingeschlossen werden. |
| Risiken / Nutzen: | Wundinfekte sind mit einer erhöhten Morbidität und Mortalität assoziiert. Einfache Massnahmen welche die Inzidenz von Wundinfekten reduzieren könnten sind somit von grossem Interesse. Die Einführung von spezialisierten Wundverschlussteams könnte die Inzidenz von Wundinfekten reduzieren da somit das primäre Operationsteam entlastet werden könnte.  Durch die Studie wird die primäre chirurgische Therapie nicht verändert. Es ist möglich, dass durch den Teamwechsel eine gewisse Unruhe und Ablenkung entsteht. Möglicherweise gibt es hierfür eine gewisse Gewöhnung im Verlauf der Studie. Teamwechsel stellen an sich nichts ungewöhnliches in der Chirurgie dar und wurden bisher nicht routinemässig sondern nur bei Bedarf durchgeführt. Für den Patienten sollte kein zusätzliches Risiko entstehen sofern nötige Information zwischen den beiden Teams weiter gegeben wird. |

Abbreviations

| ASR | Annual safety report |
| --- | --- |
| CEC | Competent Ethics Committee |
| CRF | Case Report Form |
| GCP | Good Clinical Practice |
| Ho | Null hypothesis |
| H1 | Alternative hypothesis |
| HFG | Humanforschungsgesetz (Law on human research) |
| HMG | Heilmittelgesetz |
| PI | Principal Investigator |
| SOP | Standard Operating Procedure |
| SSI | Surgical site infection |
| SNCTP | Swiss National Clinical Trials Portal |
| TMF | Trial Master File |
|  |  |
|  |  |
|  |  |
|  |  |
|  |  |
|  |  |

# STUDY ADMINISTRATIVE STRUCTURE

This trial is an investigator-initiated single-centre trial, initiated and coordinated by the Department of Visceral Surgery and Medicine, Bern University Hospital.

## Sponsor, Sponsor-Investigator

Prof. Dr. med. Guido Beldi, Department of Visceral Surgery and Medicine, University hospital Bern (Inselspital), 3010 Bern, Telephone number: 0316324820

The sponsor-investigator is responsible for the design of the study, the analysis and interpretation of data and writing of the report.

## Principal Investigator

Prof. Dr. med. Guido Beldi, Department of Visceral Surgery and Medicine, University hospital Bern (Inselspital), 3010 Bern, Telephone number: 0316328275

## Statistician ("Biostatistician")

CTU Bern, Finkenhubelweg 11

## Monitoring institution

CTU Bern, Finkenhubelweg 11

## Data Safety Monitoring Committee

A Data Monitoring Board will oversee the trial for any safety issues. A charter will be drafted before the first patient is included.

## Any other relevant Committee, Person, Organisation, Institution

None

# ETHICAL AND REGULATORY ASPECTS

The decision of the CEC concerning the conduct of the study will be made in writing to the Sponsor-Investigator before commencement of this study. The clinical study can only begin once approval from all required authorities has been received. Any additional requirements imposed by the authorities shall be implemented.

## Study registration

The study will be registered at clinicaltrials.gov. After obtaining the trial identification number the study will additional be registered at the Swiss National Clinical Trials Portal (SNCTP).

## Categorisation of study

Category A: Observational study without relation to the use of drugs or medical devices. The risk for the participant is minimal and the study provides no additional strain to the participant.

## Competent Ethics Committee (CEC)

The principal investigator ensures that the study will be approved by the local Competent Ethics Committee (Kantonale Ethikkommission Bern)

No changes are made to the protocol without prior Sponsor and CEC approval, except where necessary to eliminate apparent immediate hazards to study participants.

Premature study end or interruption of the study is reported within 15 days. The regular end of the study is reported to the CEC within 90 days, the final study report will be submitted within one year after study end.

## Ethical Conduct of the Study

The study will be carried out in accordance to the protocol and with principles enunciated in the current version of the Declaration of Helsinki, the guidelines of Good Clinical Practice (GCP) issued by ICH. The CEC will receive annual safety and interim reports and be informed about study stop/end in agreement with local requirements.

## Declaration of interest

The sponsor-investigator and the subinvestigators involved in the clinical trial declare no support from any organisation for the trial; no financial relationships with any organisations that might have an interest in the results of the trial; no other relationships or activities that could appear to have an influence on the trial.

## Patient Information

For this study no informed consent will be obtained because of the following reasons:

- Surgical site infections are assessed for surveillance according to the standards of Swissnoso. This is being performed as a routine surveillance and will not change during the study.
- The operation procedure in its technical aspect will not change during the intervention period. The only difference is another surgical team will be doing the wound closure. Therefore, the standard treatment of the patient during the intervention will not be altered compared to the control period.

An information sheet and a general consent form with explanation that we use their health-related data will be provided to all participants. Each participant will be informed that the participation in studies is voluntary and that he/she may withdraw from the general consent at any time and that withdrawal of consent will not affect his/her subsequent medical assistance and treatment.

The participant will be informed that his/her medical records may be examined by authorised individuals other than their treating physician.

The patient information sheet and the general consent form are attached (Appendix B). The formal consent of a participant, using the approved consent form, will be obtained before the participant is submitted to any study procedure.

The participant will read and consider the statement before signing and dating the general consent form, and is given a copy of the signed document. The consent form will be signed and dated by the investigator (or his designee).

## Participant privacy and confidentiality

The investigator affirms and upholds the principle of the participant's right to privacy and that they shall comply with applicable privacy laws. Especially, anonymity of the participants shall be guaranteed when presenting the data at scientific meetings or publishing them in scientific journals.

Individual subject medical information obtained as a result of this study is considered confidential and disclosure to third parties is prohibited. Subject confidentiality will be further ensured by utilising subject identification code numbers to correspond to treatment data in the computer files.

For data verification purposes, authorised representatives of the Sponsor-Investigator, the ethics committee may require direct access to parts of the medical records relevant to the study, including participants’ medical history.

## Early termination of the study

The Sponsor-Investigator may terminate the study prematurely according to certain circumstances, for example:

- ethical concerns,
- when the safety of the participants is doubtful or at risk, respectively,
- alterations in accepted clinical practice that make the continuation of a clinical trial unwise,
- early evidence of benefit or harm of the experimental intervention

## Protocol amendments

Substantial amendments are only implemented after approval of the CEC.

Under emergency circumstances, deviations from the protocol to protect the rights, safety and well-being of human subjects may proceed without prior approval of the sponsor and the CEC. Such deviations shall be documented and reported to the sponsor and the CEC as soon as possible.

All Non-substantial amendments are communicated to the CEC within the Annual Safety Report (ASR).

# Background and Rationale

## Background and Rationale

Incisional surgical site infection (SSI) is one of the most frequent complications after abdominal surgery, leading to considerable costs. we have shown that the performance of the surgical team impacts on SSI in addition to the patients pre-existing diseases ^1-3^. Pre-existing diseases of the patient mostly cannot be influenced. Therefore, we aim to optimize the performance of the surgical team in order to reduce SSI. Our data from observational studies reveal that discipline and communication within the surgical team impact on SSI^1,2,4^. With the proposed studies we now aim to address the aspect of fatigue on the performance of surgical team. The wound closure is done in a phase of the operation when concentration and motivation of the team often decreases. The exchange of the operation team (as described below in detail) represents a potential clinical applicable intervention that may improve SSI.

## Clinical Evidence to Date

We have observed that team behaviour and communication may impact on the outcome of abdominal surgery ^1,2,4^. Being the most frequent complication, surgical site infection has been used as the primary outcome parameter in these studies. The association of team behaviour and surgical site infection has been made in other surgical specialities^5-7^.

However, to alter the behaviour of the surgical team has been notoriously difficult. One attempt to impact on behaviour in the operating room was the introduction of checklists^8^. Despite being applied now in many hospitals, the success of such a measure highly depends on the education and dedication of the surgical team and failures have been described^9,10^. From team training interventions in other fields it is known that general team trainings often show limited effects^11^, whereas specific interventions are more promising ^12,13^.

With the proposed studies we now aim to overcome such behavioural risk by introducing a structural change within the process of wound closure. A specific closure team potentially should interpret the closure of the abdomen as their primary task, whereas for the first team closure has rather been a secondary task after a complex intraabdominal operation.

## Explanation for choice of comparator

The control group represents the current standard of care in which the same surgical team performs the wound closure.

In the trial group a trained team consisting of a surgeon and a student will replace the surgical team and will perform the closure of the laparotomy, irrigation of the wound and skin suturing.

We chose the design of a prospective controlled, two armed, observational study (before and after study). As the control group we will use the population of our ongoing STOP?-Study. Using this approach we have the opportunity to profit from the already established assessment of the clinical endpoint within the StOP? study that has been shown to be highly reliable.

## Risks / Benefits

The primary treatment of the patient is not affected by the trial. The change of the surgical team potentially may irritate and distract other personnel that is involved in the treatment of the patient. This may be of relevance during the initial phase of the study. The operation time may rise a little, however, a higher risk for the patient is not to be expected. Conversely, exchange of team members is frequently done in clinical routine for both surgeons and nurses.

## Justification of choice of study population

Included in the study are all patients (elective and emergency) with laparotomy and closure of the wound from Monday to Friday from 8:00 to 17:30 o clock. The time restriction is based on the difficulty to organize a team outside of regular working hours.

# STUDY OBJECTIVES

## Overall Objective

The aim of this trial is to test the impact of the exchange of the surgical team during wound closing on the incidence of SSI.

SSI is a frequent complication after laparotomy. Its incidence depends of the performance of the surgical team. Evidence revealed however, that the performance of the surgical team may be reduced at the end of the surgical procedure because of fatigue. By the exchange of surgical team members high quality of surgical performance may be maintained.

## Primary Objective

To assess the influence of a specialised wound closure team on the incidence of SSI 30 days after the operation.

## Secondary Objectives

To assess the influence of a specialised wound closure team on mortality, fascial dehiscence.and postoperative complications according to Clavien-Dindo.

# STUDY OUTCOMES

## Primary Outcome

SSI that occur after surgery will be assessed according to the criteria developed by the Centers for Disease Control and Prevention^14^. Infections will be categorized as incisional (superficial or deep) infections or organ–space infections. Superficial SSI (type 1) involve only skin and subcutaneous tissueand exclude stitch abscesses. Deep SSI (type 2) involve deeper soft tissues, like fascia and muscle, at the site of incision. Organ–space SSI (type 3) involve any organ or body cavity.

Incidence of SSI is assessed according to the clinical routine, following the established protocol of Swissnoso. Data collection according to the Swissnoso protocol will be extended to all surgeries included. The protocol includes direct contact to the patient:

- All patients receive a standardised follow-up phone call at least 30 days after the operation to assess incidence of SSIs and other complications.
- If patients do not respond to follow-up phone calls, five documented attempts to contact the patients followed by rapid contacts with the subject’s general practitioner or other medical staff involved in the medical treatment of the patients, will be performed before loss of follow-up is documented.

Surveillance according to Swissnoso is performed by the department of infectious diseases of the University hospital of Bern and are further supervised by external reviewers from Swissnoso. Therefore the observers are independent and evaluation bias is minimized.

## Secondary Outcomes

The assessment of postoperative mortality will be included to the telephone interview and assessed as follows:

Mortality at 30 days: Observer receives the information during the follow-up phone call that the patient died. If patients do not respond to follow-up phone calls, five documented attempts to contact the patients followed by rapid contacts with the subject’s general practitioner or other medical staff involved in the medical treatment of the patients, will be performed before loss to follow-up is documented. In addition, the Zivilstandsamt will be contacted once 3 months after surgery.

Postoperative fascial dehiscence at 30 days postoperative.

Complications: Assessment according to Clavien-Dindo grading

## Other Outcomes of Interest

In order to perform subgroup analyses or adjusted analyses, the following variables are assessed (Dokument 1 Swissnoso):

- - Type of operation (Haupteingriff, Sekundäreingriff)
  - Grade of contamination (Kontaminationsgrad)
  - Duration of the procedure
  - Height and weight of the patient.

# STUDY DESIGN

## General study design and justification of design

Prospective controlled, two armed, observational, monocentric study (before and after study).

To evaluate the impact of a specialised wound-closing team on surgical site infections

Included in the study are all patients (over 18 years, elective and emergency) with laparotomy and closure of the wound from Monday to Friday from 8:00 to 17:30 o’clock. We aim to include a total of 1785 participants.

The control group consists of the study population of the StOP?-study. In this population the wound closure was done by the same operation team under supervision of the main surgeon.

The screening of the first participant in the control group started on the 01.03.2016 and ended with the STOP? study on the 30.11.2016. The inclusion of patients in the intervention group takes place between 1.3.2017 and 30.11.2017. Therefore the end of data collection will be the 31.12.2017.

## Methods of minimising bias

### Randomisation

This study will not be randomized.

### Other methods of minimising bias

In this study all patients within two periods will be included thereby limiting selection bias. However, it is to be expected that the incidence of surgical site infection may decrease over time in response to other measures. Such gradual decrease is expected of occur in both periods and may be corrected.

# STUDY POPULATION

## Eligibility criteria

Participants fulfilling all of the following inclusion criteria are eligible for the study:

- General Consent as documented by signature (Appendix B)
- Patients undergoing elective or emergency laparotomy from Monday to Friday with wound closure from 8:00 until 17:30 and duration of operation.
- Age over 18 years
- Any indication for laparotomy

The presence of any one of the following exclusion criteria will lead to exclusion of the participant:

- Patients < 18 years of age
- Patients with preexisting SSI

## Recruitment and screening

All patients with documented general consent undergoing elective or emergency laparotomy will be included in this study.

## Assignment to study groups

The patients will be assigned to the study groups according to the time point of inclusion. In the period between 1.3.2016 to 30.11.2016 the control group was assessed. Patients operated between 1.3.2017 and 30.11.2017 represent the intervention group.

## Criteria for withdrawal / discontinuation of participants

Not applicable.

# STUDY INTERVENTION

## Identity of Intervention

### Experimental Intervention

The intervention consists of the exchange of the primary surgical team with a second surgical team that consists of one surgeon and one student. The first surgical team then may leave the operation theatre but is continuously accessible for questions.

### Control Intervention

In the control group the abdomen is closed according the clinical routine by the primary surgical team.

The closure in both groups will be identical and consists of a running PDS suture and skin closure according to the surgeons preference.

## Compliance with study intervention

Questionnaires will be filled out by the circulating nurse in the operating theatre in order to identify if the exchange of the surgical team has been performed and what team members were exchanged.

## Data Collection and Follow-up for withdrawn participants

Information of patients that withdraw the general consent will be stored anonymously.

## Trial specific preventive measures

Not applicable.

## Concomitant Interventions (treatments)

The study intervention does not interfere with the medical treatment. Therefore concomitant interventions for other studies may be performed.

# STUDY ASSESSMENTS

## Assessments of outcomes

### Assessment of primary outcome

Surgical site infections (SSI) that occur after surgery will be assessed according to the criteria developed by the Centers for Disease Control and Prevention. Infections will be categorized as incisional (superficial or deep) infections or organ–space infections. Superficial SSI (type 1) involve only skin and subcutaneous tissue and exclude stitch abscesses. Deep SSI (type 2) involve deeper soft tissues, like fascia and muscle, at the site of incision. Organ–space SSI (type 3) involves any organ or space.

Incidence of SSI is assessed according to the clinical routine, following the established protocol of Swissnoso. Data collection according to the Swissnoso protocol will be extended to all surgeries included. The protocol includes direct contact to the patient:

- All patients receive a standardised follow-up phone call at least 30 days after the operation to assess incidence of SSIs and other complications.
- If patients do not respond to follow-up phone calls, five documented attempts to contact the patients followed by rapid contacts with the subject’s general practitioner or other medical staff involved in the medical treatment of the patients, will be performed before loss of follow-up is documented.

### Assessment of secondary outcomes

Postoperative mortality

The assessment of postoperative mortality will be included into the telephone interview and assessed as follows:

Mortality at 30 days: Observer receives the information from the postoperative stay that the patient died

Complications: Assessment according to Clavien-Dindo Grading

Postoperative fascial dehiscence at 30 days postoperative: As reported in prior reports / operation reports.

Observer receives the information during the follow-up phone call that the patient died. If patients do not respond to follow-up phone calls, five documented attempts to contact the patients followed by rapid contacts with the subject’s general practitioner or other medical staff involved in the medical treatment of the patients, will be performed before loss of follow-up is documented. In addition, the Zivilstandsamt will be contacted once 3 months after surgery.

### Assessment of other outcomes of interest

In order to perform subgroup analyses or adjusted analyses, following variables are assessed:

- Type of operation (primary, secondary surgery)
- Grade of contamination
- Duration of the procedure
- Height and weight of the patient
- Comorbidities

### Assessment of safety outcomes

As the standard treatment will not change in this study, any deviation from the standard treatment that may be related to the intervention will be recorded.

# STATISTICAL METHODS

## Hypothesis

Null Hypothesis: The introduction of a trained closure team does not change the incidence of SSI after abdominal surgery

Alternative Hypotheses: The introduction of a trained closure team changes the incidence of SSI after abdominal surgery.

## Determination of Sample Size

Sample size: The incidence of SSI after abdominal surgery in the department for Visceral Surgery and Medicine is 20%. The aim is to detect a reduction of the overall incidence rate of SSI from 20% to 14% after a follow-up of 30 days. We defined the level of significance at 5% and power of 80%, assuming normal approximation to binomial distribution requires 484 surgeries in each cell, thus 968 patients overall. In the ongoing StOP?-study we have a drop out and study refusal rate of maximal 20%. Thus, a total of 1160 participants (580 per arm) need to be included.

Duration: The average number of laparotomies from 8:00 AM to 5:30 PM from Monday to Friday per year are 720. The study schedule includes first 9 months in the control (baseline: 2^nd^ arm of the STOP? Study) and then 9 months in the intervention group.

## Statistical criteria of termination of trial

No interim analysis is foreseen.

## Planned Analyses

### Primary Analysis

The primary outcome parameter will be the comparison of incidence of SSI rates before and after the intervention. An interrupted time series design will be used, including 36 data points (based on about 20 surgeries per data point) before and about 36 data points (based on about 20 surgeries per data point) after the intervention. Interrupted time series allow to assess influences of trends and random fluctuations over time. We will include type of surgery, the NNIS-risk score (composed of ASA-score, wound contamination and duration of the surgery), as well as patient age as covariates. Covariates are included to adjust for chance imbalances between baseline and post intervention groups.

### Secondary Analyses

Interrupted time series analyses will also be performed for the secondary outcome parameters (mortality and complication rate), including the same covariates as for the primary outcome parameter.

There are many known influences on SSI^15^. Given the extensive inclusion criteria (basically all surgeries except those where SSI was the reason for surgery), we expect a similar risk profile before and after the intervention. The Swissnoso protocol includes assessing ASA-score, duration of surgery, wound contamination class and type of surgery. This information allows to calculate the NNIS-risk score for each surgery.

# QUALITY ASSURANCE AND CONTROL

## Data handling and record keeping / archiving

### Case Report Forms

All protocol required procedures along with information necessary to report the observations and tests described in this protocol will be recorded online and as hard copy by the department of Visceral Surgery and Medicine, Bern University hospital. All data entered on the case report form (CRF) must be documented in a source document with all the information on which the entries in the CRF are based being available in the patient files e. g. results of laboratory investigations. The case report form is not a source document. The clinical investigator is responsible for the identity of the data in the patient file and the correct entry of the data into the CRF. The investigator must review all pages within the CRF for accuracy and consistency with the protocol, and sign and date the CRF sign-off page(s) upon completion.

After prior agreement, a check of the consistency of data between the patient files, raw data and CRF as well as with other documents related to the study may be conducted by the responsible authorities and/or by monitors (inspection/audit/monitoring).

### Data security and back-up

The redcap database that has been used for the STOP study will be used for this trial. Authorized users have access to the database using a personal password. A role concept (site investigator, statistician, monitor, administrator etc.) regulates permission for each user to use the database as he/she requires.

### Analysis and archiving

At final analyses, data files will be extracted from the database into statistical packages to be analysed. The status of the database at this time is recorded in special archive tables. These tables cannot be altered in future. The study database with all archive tables will be securely stored by Inselspital Bern for at least 10 years.

### Record keeping

A copy of the final completed CRFs is retained by the investigator, who must ensure that is it stored with other study documents, such as the protocol, the investigator`s brochure, the trial master file and any protocol amendments.

### Data validation

A multilevel data validation plan was conceived in order to guarantee the correctness and consistency of the data. Data can be entered only after a check of completeness and plausibility. Furthermore, data are cross-checked for plausibility with previously entered data for that participant. Retrospective alterations of data are recorded in an audit table. Besides time, data field, original value and altered value, the person and reason for alteration are also recorded. A pseudonym forms the participant key automatically generated by the database with a secure hash algorithm compliant to data protection regulations. The algorithm uses the following information to generate the pseudonym: first name, last name, birth name, birth date, gender. These data are at no time stored in the database. Exchange of data takes place exclusively by participant key.

## Monitoring and supervision procedures and data quality assurance

### Monitoring

In order to guarantee a high quality of the study and data retrieval, on-site monitoring will be performed by CTU Bern, Finkenhubelweg 11. Data protection rights will be respected. Before study start (first participant in) a monitoring plan detailing all monitoring related procedures will be developed.

### Supervision

In order to guarantee the identification of the primary endpoint, supervision will be performed by an independent physician as it is foreseen by Swissnoso. At the time of submission, this independent physician is PD. Dr. Jonas Marschall, member of the infection prevention team of the Inselspital.

## Publication policy and final report

The results of the study will be published via oral communications during national and international meetings and via written publications. The final report will present the results of the study, including appropriate tables and figures in the spirit of an unbiased objectivity. The principle investigator will provide the IRB/IEC with a summary of the study's findings, and if applicable the regulatory authorities with any reports required. The report or parts of it may be submitted in the form of a summary, a synopsis, published article or in some other similar way. Senior author will be Guido Beldi. Co-authorship on any of the publications will be based on conceptual contribution to the study according to the criteria of the International Committee of Medical Journal Editors. The heads and one additional co-author of the participating institutions will be listed in alphabetical order.

# INSURANCE

Insurance will be provided by the Sponsor.

# REFERENCES

1. Beldi G, Bisch-Knaden S, Banz V, Muhlemann K, Candinas D. Impact of intraoperative behavior on surgical site infections. *American journal of surgery.* 2009;198(2):157-162.

2. Tschan F, Seelandt JC, Keller S, et al. Impact of case-relevant and case-irrelevant communication within the surgical team on surgical-site infection. *The British journal of surgery.* 2015;102(13):1718-1725.

3. Kurmann A, Peter M, Tschan F, Muhlemann K, Candinas D, Beldi G. Adverse effect of noise in the operating theatre on surgical-site infection. *The British journal of surgery.* 2011;98(7):1021-1025.

4. Kurmann A, Peter M, Tschan F, Muhlemann K, Candinas D, Beldi G. Adverse effect of noise in the operating theatre on surgical-site infection. *The British journal of surgery.*98(7):1021-1025.

5. Fan CJ, Pawlik TM, Daniels T, et al. Association of Safety Culture with Surgical Site Infection Outcomes. *J Am Coll Surg.* 2016;222(2):122-128.

6. Gfrerer L, Mattos D, Mastroianni M, et al. Assessment of patient factors, surgeons, and surgeon teams in immediate implant-based breast reconstruction outcomes. *Plastic and reconstructive surgery.* 2015;135(2):245e-252e.

7. Umit UM, Sina M, Ferhat Y, Yasemin P, Meltem K, Ozdemir AA. Surgeon behavior and knowledge on hand scrub and skin antisepsis in the operating room. *J Surg Educ.* 2014;71(2):241-245.

8. Haynes AB, Weiser TG, Berry WR, et al. A surgical safety checklist to reduce morbidity and mortality in a global population. *N Engl J Med.* 2009;360(5):491-499.

9. Urbach DR, Govindarajan A, Saskin R, Wilton AS, Baxter NN. Introduction of surgical safety checklists in Ontario, Canada. *N Engl J Med.* 2014;370(11):1029-1038.

10. Durando P, Bassetti M, Orengo G, et al. Adherence to international and national recommendations for the prevention of surgical site infections in Italy: results from an observational prospective study in elective surgery. *Am J Infect Control.* 2012;40(10):969-972.

11. Nielsen PE, Goldman MB, Shapiro DE, Sachs BP. Effects of teamwork training on adverse outcomes and process of care in labor and delivery: A randomized controlled trial. *Obstetrics and Gynecology.* 2007;109(6):1458-1458.

12. Weaver SJ, Rosen MA, DiazGranados D, et al. Does teamwork improve performance in the operating room? A multilevel evaluation. *Jt Comm J Qual Patient Saf.* 2010;36(3):133-142.

13. Salas E, DiazGranados D, Weaver SJ, King H. Does team training work? Principles for health care. *Acad Emerg Med.* 2008;15(11):1002-1009.

14. National Nosocomial Infections Surveillance (NNIS) System Report, data summary from January 1992 through June 2004, issued October 2004. *Am J Infect Control.* 2004;32(8):470-485.

15. Mangram AJ, Horan TC, Pearson ML, Silver LC, Jarvis WR. Guideline for prevention of surgical site infection, 1999. Centers for Disease Control and Prevention (CDC) hospital infection control practices advisory committee. *Am J Infect Control.* 1999;27(2):97-132.

# Appendix

## Swissnoso CRF


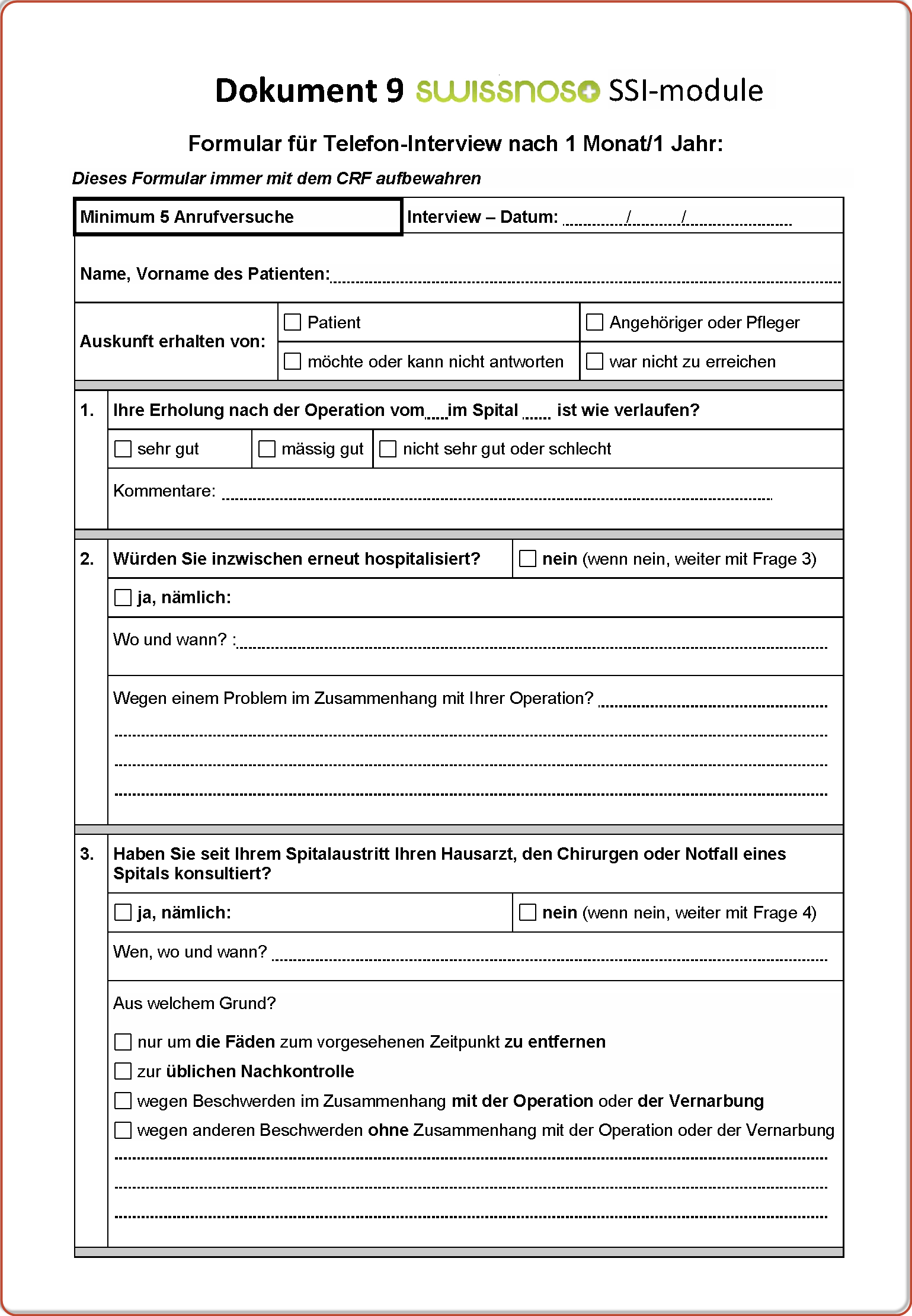

## Patience Information (D,F,E,I)

## General Consent (D,F,E,I)
